# Effects of a Web-based Weight Management Education Program on Various Factors for Overweight and Obese Women: Randomized Controlled Trial

**DOI:** 10.2196/42402

**Published:** 2024-04-18

**Authors:** Yunmin Han, Hoyong Sung, Geonhui Kim, Yeun Ryu, Jiyeon Yoon, Yeon Soo Kim

**Affiliations:** 1 Department of Physical Education Seoul National University Seoul Republic of Korea; 2 Department of Military Kinesiology Korea Military Academy Seoul Republic of Korea; 3 Department of Aviation Sports Korea Air Force Academy Cheongju Republic of Korea; 4 Department of Kinesiology Iowa State University Ames, IA United States

**Keywords:** weight loss, obesity, health education, self-management, health promotion, tailored feedback, web-based intervention, behavior change

## Abstract

**Background:**

Mediated diet and exercise methods yield effective short-term weight loss but are costly and hard to manage. However, web-based programs can serve many participants, offering ease of access and cost-efficiency.

**Objective:**

This study aimed to compare the effectiveness of a web-based weight management program through web-based education alone (MINE) or combined with tailored video feedback (MINE Plus) with a control (CO) group.

**Methods:**

This intervention included 60 Korean women with overweight and obesity (BMI≥23 kg/m^2^) aged 19 years to 39 years old. We randomly allocated 60 participants to each of 3 groups: (1) MINE group (web-based education video and self-monitoring app), (2) MINE Plus group (web-based education video, self-monitoring app, and 1:1 tailored video feedback), and (3) CO group (only self-monitoring app). Web-based education included nutrition, physical activity, psychological factors, medical knowledge for weight loss, goal setting, and cognitive and behavioral strategies. Tailored feedback aimed to motivate and provide solutions via weekly 10-minute real-time video sessions. The intervention lasted 6 weeks, followed by a 6-week observation period to assess the education's lasting effects, with evaluations at baseline, 6 weeks, and 12 weeks. A generalized linear mixed model was used to evaluate time and group interactions.

**Results:**

In the intention-to-treat analysis including all 60 participants, there were significant differences in weight change at 6 weeks in the MINE and MINE Plus groups, with mean weight changes of –0.74 (SD 1.96) kg (*P*=.03) and –1.87 (SD 1.8) kg (*P*<.001), respectively, while no significant change was observed in the CO group, who had a mean weight increase of 0.03 (SD 1.68) kg (*P*=.91). After 12 weeks, changes in body weight were –1.65 (SD 2.64) kg in the MINE group, –1.59 (SD 2.79) kg in the MINE Plus group, and 0.43 (SD 1.42) kg in the CO group. There was a significant difference between the MINE and MINE Plus groups (*P*<.001). Significant group × time effects were found for body weight in the MINE and CO groups (*P*<.001) and in the MINE Plus and CO groups (*P*<.001), comparing baseline and 12 weeks. Regarding physical activity and psychological factors, only body shape satisfaction and health self-efficacy were associated with improvements in the MINE and MINE Plus groups (*P*<.001).

**Conclusions:**

This study found that the group receiving education and tailored feedback showed significant weight loss and improvements in several psychological factors, though there were differences in the sustainability of the effects.

**Trial Registration:**

Korea Disease Control and Prevention Agency (KDCA) KCT0007780: https://cris.nih.go.kr/cris/search/detailSearch.do/22861

## Introduction

Obesity is linked to a wide range of diseases and increases the risk of morbidity and mortality [1]. In South Korea, there has been a steady yearly increase in the obesity trend, from 29.7% in 2009 to 36.3% in 2019 [2]. The social and economic burdens of obesity on health care systems worldwide are significant, with costs associated with obesity-related diseases continuing to rise [3]. Addressing obesity effectively not only improves individual health outcomes but also reduces these broader economic impacts [4]. As a result, this trend has led to a growing medical burden, which has become a significant social issue [5]. It has become an ongoing public health concern due to its increasing prevalence each year [6]. Furthermore, it is associated with a high risk of metabolic syndrome and chronic diseases such as type 2 diabetes, high blood pressure, and cardiovascular disease [7,8]. Studies have shown that people with obesity have a lower quality of life than those who do not [9], causing mental health problems such as an increased risk of depression [10]. Therefore, maintaining a healthy weight is essential to prevent physical and psychological health problems.

Treatments for obesity encompass various strategies, including psychological management. Research has shown that behavior modification programs targeting weight loss not only lead to significant weight reduction but also a decrease in depression [11]. Furthermore, studies focusing on the psychological aspects of weight loss, such as depression, anxiety, and quality of life, reveal that women with overweight or obesity are more likely to view their body image pessimistically than men [12,13]. Addressing these psychological challenges through obesity education and management programs grounded in social cognitive theory has been proven effective [14].

Over the years, various weight loss programs, including behavioral therapies, have been carried out to address obesity [15,16]. Exercise and nutrition are usually addressed by experts face to face in most conventional weight loss studies. Recently, web-based research has become increasingly popular due to its ability to save time, costs, and human resources compared with previous face-to-face research. However, these studies have several limitations, such as participants needing more motivation, data collection problems, and low attrition rates [17]. The sustainability of health behaviors postintervention is critical for long-term weight management success. Exploring strategies to maintain and support these behaviors beyond the intervention period is essential, underscoring the importance of follow-up and continued engagement [18]. Moreover, digital transformation in the exercise and medical sectors has become increasingly inevitable due to advancements in artificial intelligence and digital health care. The digital era has accelerated the growth of the online home training and telemedicine market [19]. Therefore, more studies are needed to increase the sample size, include various target groups, and confirm continuous effects to overcome the limitations.

According to a meta-analysis of behavioral change programs, study duration has varied from 2 weeks to 78 weeks (mean 26 weeks). However, given that only 5 of the 35 digital-based studies included a follow-up period, the number of studies with such periods was very limited [20]. Furthermore, studies that did not include goal setting and feedback showed low research quality. Therefore, to emphasize the effectiveness of behavioral change programs, high-quality research with feedback and goal setting is needed [21].

The primary aim of this study was to compare the weight loss effects of web-based education and feedback among 3 groups (2 interventions and 1 control) over a given period and to determine whether participants could implement what they learned and achieve weight loss. The second aim was to compare whether the effects of physical activity and psychological factors persisted in these groups during the observation period.

## Methods

### Recruitment and Participants

Participants in this study were recruited from Seoul National University students and staff members through advertisements (eg, email, flyers, and social media). The advertisements included information on the purpose of the study, data collection methods, and benefits offered to participants. According to the Korean Society for the Study of Obesity, a BMI of 23 kg/m^2^ to 24.9 kg/m^2^ is considered overweight, a BMI of 25 kg/m^2^ to 29.9 kg/m^2^ is considered first-degree obesity, a BMI of 30 kg/m^2^ to 34.9 kg/m^2^ is considered second-degree obesity, and a BMI ≥35 kg/m^2^ is defined as severe obesity [22]. Eligible participants were young women aged 19 years to 39 years, with a BMI >23 kg/m^2^ according to Asian standards, who were able to listen to and write Korean, and who could use the Internet and smartphone devices.

Exclusion criteria were a loss of more than 10% of body weight in the past 6 months, previous obesity surgery, and pregnancy. People diagnosed with severe mental illness or cardiovascular metabolism or who were receiving medication that could affect weight loss were also excluded. All participants submitted their informed consent before enrollment.

### Interventions

**Figure 1:**
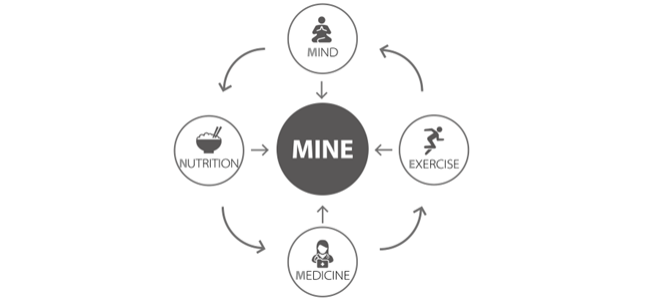
Program curriculum characteristics including research and methods related to nutrition, psychology, exercise, and medicine for weight management.

The web-based program carried out in this study is referred to as “MINE,” which is a combination of “Mind,” “Medicine,” “Nutrition,” and “Exercise.” Participants received necessary education on weight loss in all 4 of these fields (Figure 1).

**Figure 2:**
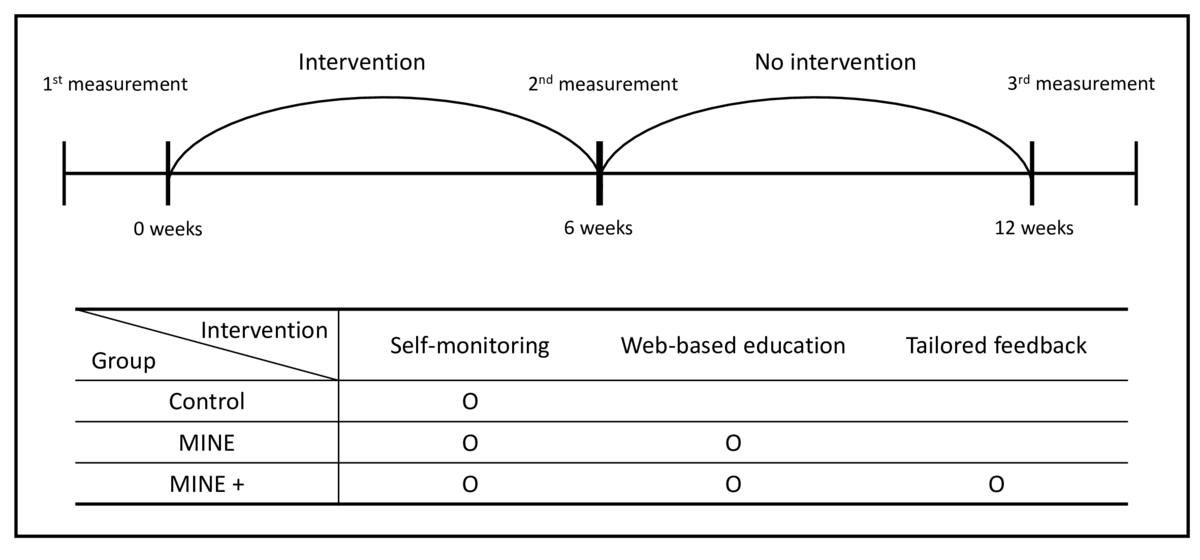
Experimental period and group type.

This program does not force physical activity on the participants nor interfere with their diet. Instead, through education and feedback as the intervention, it aims to change behavior to achieve weight loss by empowering the participants to make better choices on their own. The group that received only web-based education was called “MINE,” the group that received web-based education and tailored feedback was called “MINE Plus,” and the waiting list group was called “Control” (Figure 2).

The sample size was calculated using GPower version 3.1. The significance level was .05, the effect size was 0.25, and the power was 0.8. The minimum number of participants was 42, the dropout rate was set at approximately 20% based on previous studies [23], and the number of participants was set at 60 and randomly assigned to 3 groups. We asked the participants to send their self-monitoring records once a week. However, there was no compulsion to send data even if the data were insufficient. The behavior change strategy was composed by incorporating the basic concepts of personalized cognitive behavioral therapy for obesity that were previously researched, as well as goal setting, social support, action planning, coping planning, and self-monitoring, which are frequently used in eHealth interventions. This information was integrated and utilized in conjunction with the education content and feedback [24,25]. The education content was divided into exercise, nutrition, psychology, and medical areas to establish knowledge and behavioral change strategies necessary for weight loss. Educational materials were produced by referring to previous scientifically proven studies and were verified by qualified experts in the field. The web-based education videos lasted 15 minutes to 20 minutes; details are shown in Table 1. In addition, these videos were delivered via video links and materials for each week through chat rooms organized by the groups.

**Table 1:** Educational content order and topics.

Number	Educational sessions	Field
1	Setting my diet	Nutrient
2	Setting my workout	Exercise
3	How to control my appetite	Psychology
4	Understanding fat burning system	Medicine
5	Healthy nutrients vs worst nutrients	Nutrient
6	Understanding the benefits of exercise	Exercise
7	Dealing with other people’s perceptions and getting rid of stress	Psychology
8	Understanding the digestive system	Medicine
9	Practical diet recipes	Nutrient
10	How to exercise at home or the workplace	Exercise
11	How to deal with eating out and appointments	Psychology
12	Understanding the endocrine system and metabolism related to weight control	Medicine
13	Making your own sustainable diet plan	Nutrient
14	Making your own sustainable exercise goals	Exercise
15	Establishing long-term weight management strategies based on social cognitive theory	Psychology
16	Developing a self-check list and a plan for improving health indicators	Medicine

The feedback was based on the protocol of the web-based weight loss program called “POWeR” developed by Dennison et al [26] and a social cognitive theory strategy for obesity treatment developed by Dalle Grave et al [24]. Before proceeding with the feedback, basic lifestyle and weight loss experience information was collected through an online survey. The survey included information such as usual mealtimes, meal volume, sleep, health information, weight loss history, difficulties with losing weight, and personal goals. The feedback schedule was then implemented by entering the ID at the desired time through the online form and entering the Zoom video conference at the indicated time. Coaching sessions were conducted once a week, lasting approximately 15 minutes to 20 minutes each. Both groups (MINE and MINE Plus) received weekly educational materials and alarm messages about watching and practicing educational videos through group chat rooms. After receiving education, it was considered that the education was completed by submitting quizzes related to the content. In addition, a smart electronic weighing scale was provided as a research incentive. Of the 2 groups who did not receive feedback during the observation period, the MINE group received feedback, and the control group received educational videos and feedback after the end of the follow-up period.

### Outcomes

The study lasted for a total of 12 weeks, consisting of a 6-week intervention period followed by a 6-week follow-up period. Participants' demographic data included age, marital status, and education level. The main outcome was to confirm the results related to body weight change at 6 weeks and 12 weeks after baseline. The secondary outcome was to confirm the level of physical activity, eating attitudes, satisfaction with body shape, and health self-efficacy.

The participants were examined in the laboratory after signing a consent form. Height, body weight, body composition, waist circumference, and blood pressure (BP) were measured. Height was measured using a BSM230 (Biospace), and weight and body fat were measured using an InBody 720 (Biospace). Participants were advised to avoid heavy meals, water, and intense physical activity for at least 2 hours before undergoing the InBody measurement to minimize potential measurement bias. Waist circumference was marked in centimeters using a tape measure from the middle of the lower rib to the upper iliac. For the BP measurement, an arm cuff for adults was placed around the left upper arm (Watch BP 03, Microlife AG). After measuring BP twice at rest, the average of the 2 values was calculated for the final BP index. We conducted 4 surveys after collecting the basic physical information. Physical activity was measured using the Global Physical Activity Questionnaire (GPAQ) and calculated as metabolic equivalents (METs) [27]. In addition, the Eating Attitudes Test-26 (EAT-26), developed by Garner and Garfinkel [28], was used to measure attitudes toward eating, while the Body Shape Questionnaire (BSQ), developed by Cooper et al [29], was used to measure satisfaction with body shape. In addition, the Self-Rated Abilities for Health Practices (SRAHP) scale developed by Becker et al [30] was used as a measure of health self-efficacy [30]. Validity and reliability were verified for all questionnaires translated into Korean [31-33].

The EAT score was obtained by subtracting 65 points from the total score. A tendency for eating problems is assumed if the final score is 18 or higher, while a severe eating problem is considered to exist if the final score is 25 or higher [33]. The BSQ is a 32-point to 192-point scale, with higher scores indicating a greater sense of obesity and lower overall satisfaction with body shape [29,34]. The SRAHP is a 24-point to 96-point scale, with higher scores indicating a greater sense of self-efficacy regarding one's health [30,31]. After the measurements were completed, participants were randomly allocated to the groups through a lottery. Precautions for participation in the study were delivered orally.

The same methods and questionnaires were used for every measurement. In addition, a program satisfaction survey on web-based education programs was conducted, and tailored feedback was provided on the intermediate measurement after the intervention. The survey included satisfaction with the overall program, satisfaction with personal feedback, and recommendations for the program (see Multimedia Appendix 1); it was modified by referring to the satisfaction survey in the preliminary study developed by Yip et al [35]. 

### Statistical Analysis

Intention-to-treat analysis was performed for all outcomes at 6 weeks and 12 weeks. To determine differences in the variables, we set the time (baseline, 6 weeks, 12 weeks) as the repeated factors for body weight, body composition, blood pressure, physical activity, mental health score, and physical health score. 

The Shapiro-Wilk normality test for each group was performed. Only the MINE Plus group did not show a normal distribution (*P*<.01). Therefore, for variables that did not have a normal distribution, such as body weight, BMI, waist circumference, and physical activity, a generalized linear mixed model was used to fit log-transformed data [36]. A linear mixed model method was used for the remaining variables satisfying the normality test [37]. These methods set fixed and random effects, and the interaction effect of time and group on the outcome was confirmed as outcomes of the variables. All statistical analyses were conducted using R studio (version 4.0.3). The mean and SD were calculated using descriptive statistical analysis, and the significance level was set at *P*<.05.

### Ethical Considerations

The study received approval from the international review board at Seoul National University, Seoul, South Korea (SNU IRB NO. 2109/002-007). All participants provided written consent to participate, with a clear process established for medical referral and reporting any potential harm arising from their participation. The research also emphasized privacy and confidentiality protection for human subjects by ensuring that all study data were either anonymous or de-identified, accompanied by a brief description of the protective measures in place. To safeguard the privacy and confidentiality of participants, additional details on these protections were provided. As compensation for their involvement, participants received a smart scale valued at approximately US $20.

## Results

### Participant Characteristics

**Figure 3:**
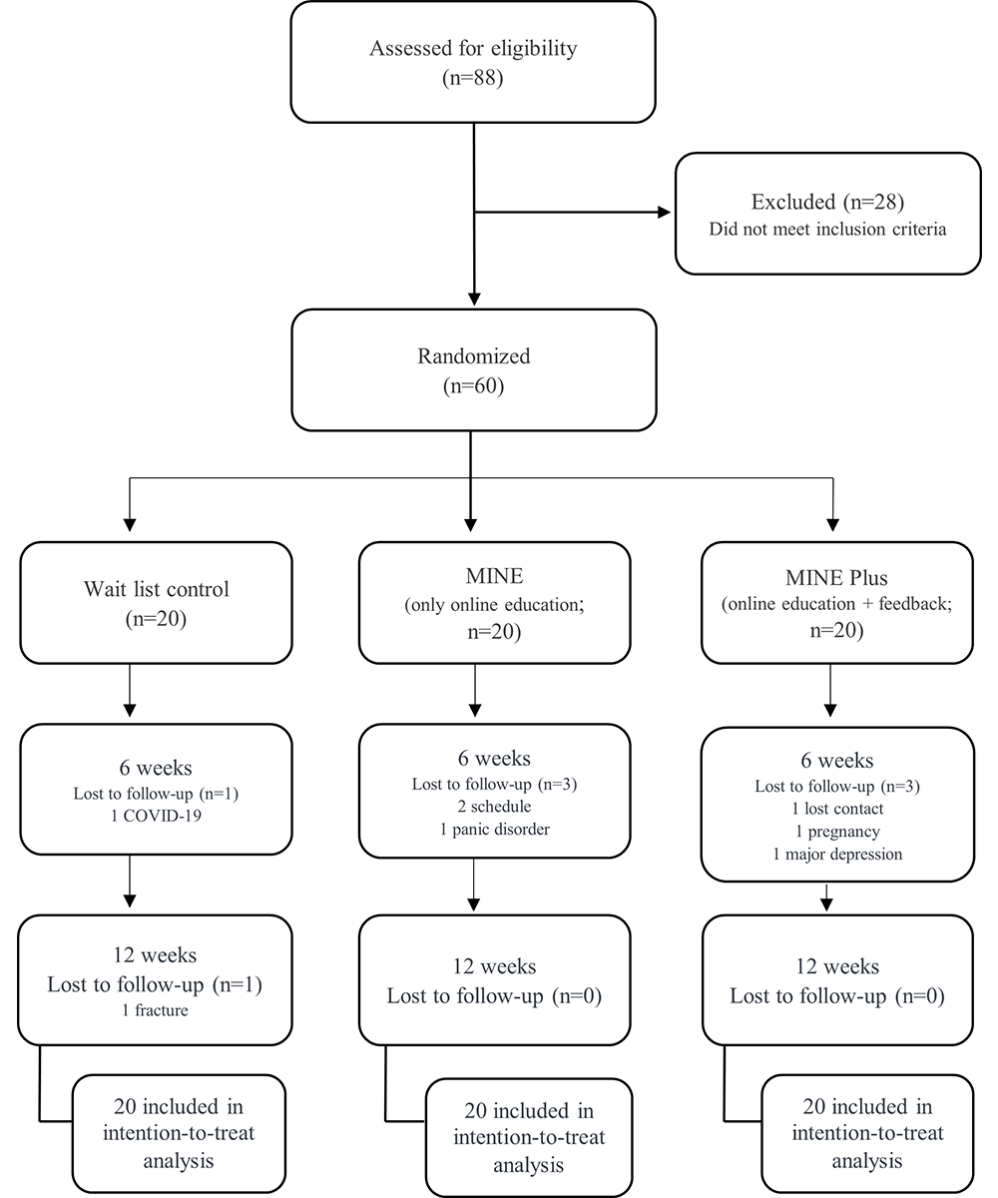
Flow diagram of participants through the trial.

Of the 88 participants, 28 were excluded because their BMI did not exceed 23 kg/m^2^ or they did not meet the inclusion criteria (Figure 3). A total of 60 people were assigned to the 3 groups, 20 per group, through random allocation; for their characteristics, see Table 2. All 60 participants were analyzed using the intention-to-treat method. In addition, missing values for unmeasured participants and participants who dropped out were analyzed as the last measured data through the last observation carried forward method [38].

**Table 2:** Baseline characteristics of participants (N=60).

Characteristic		MINE^a^ (n=20), mean (SD)	MINE Plus^b^ (n=20), mean (SD)	Control (n=20), mean (SD)
Age (years)		29.7 (5.44)	28.2 (3.75)	30.3 (5.06)
Weight (kg)		65.82 (7.23)	70.64 (13.07)	66.2 (7.08)
BMI (kg/m^2^)		25.3 (2.09)	26.5 (4.11)	25.2 (2.00)
Body fat percentage (%)		36.8 (4.59)	38.5 (5.98)	36.5 (4.68)
Waist circumference (cm)		84.0 (6.21)	86.9 (9.81)	84.6 (6.54)
**Blood pressure (mm Hg**)				
	Systolic	117 (10.63)	114 (7.98)	117 (10.98)
	Diastolic	74 (8.03)	72 (6.44)	76 (10.44)
Resting heart rate (bpm)		78 (13.22)	75 (10.31)	82 (11.29)
Physical activity (METs^c^/week)		1717 (1368)	1246 (1226)	1853 (2153)
EAT-26^d^		6.79 (16.84)	10.53 (12.64)	6.84 (17.89)
BSQ^e^		110.55 (32.96)	116.63 (26.48)	118.79 (27.56)
SRAHP^f^		70.88 (12.53)	74.02 (11.74)	72.88 (10.66)

^a^MINE: only online education.

^b^MINE Plus: online education + tailored feedback.

^c^MET: metabolic equivalent.

^d^EAT-26: Eating Attitudes Test-26 (Korean version).

^e^BSQ: Body Shape Questionnaire (Korean version).

^f^SRAHP: Self-Rated Abilities of Health Practices (Korean version).

### Weight Loss

**Figure 4:**
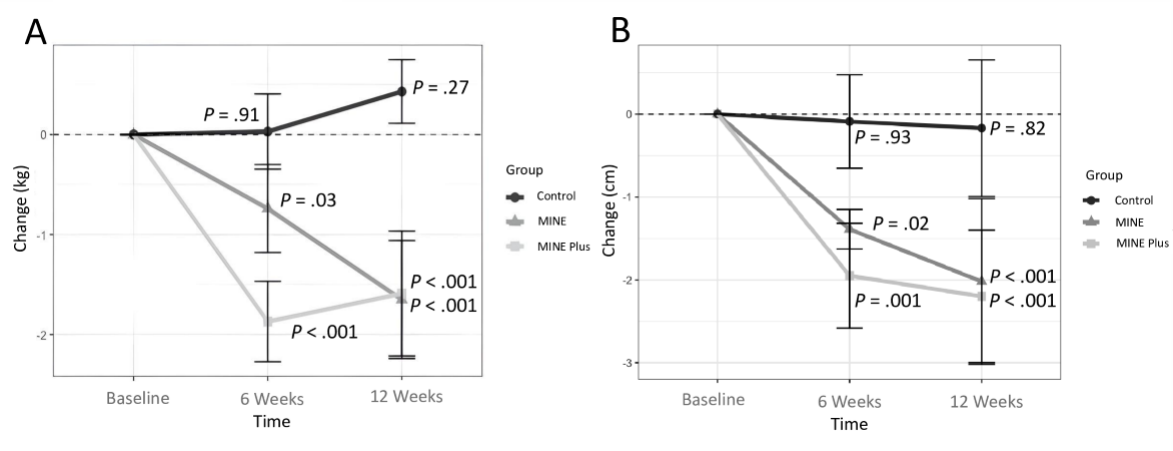
Mean changes in (A) body weight and (B) waist circumference at baseline, 6 weeks, and 12 weeks, with *P* values for differences from baseline at 6 weeks and 12 weeks. MINE: only online education; MINE Plus: online education + tailored feedback.

From baseline to week 6, the MINE group showed a significant mean weight reduction of –0.74 (SD 1.96) kg (*P*=.03), the MINE Plus group showed a significant mean weight reduction of –1.87 (SD 1.8) kg (*P*<.001), and the control group showed a mean weight increase of 0.03 (SD 1.68) kg (*P*=.91; Figure 4). From week 6 to week 12, the mean weight reduction was –0.91 (SD 2.2) kg (*P*<.001) for the MINE group, while the mean increases in weight were 0.28 (SD 1.72) kg (*P*=.24) for the MINE Plus group and 0.41 (SD 1.42) kg (*P*=.23) for the control group. Comparing the baseline with week 12, the MINE group showed a total mean reduction of –1.65 (SD 2.64) kg (*P*<.001), while the MINE Plus group showed a significant mean weight reduction of –1.59 (SD 2.79) kg (*P*<.001). In contrast, the control group showed a mean weight increase of 0.43 (SD 1.42) kg (*P*=.27). From baseline to Week 12, waist circumference significantly decreased by a mean –2.02 (SD 4.47) cm (*P*<.001) in the MINE group and –2.2 (SD 3.58) cm (*P*<.001) in the MINE Plus group, but the decrease of a mean –0.17 (SD 3.72) cm in the control group was not significant (*P*=.82).

**Figure 5:**
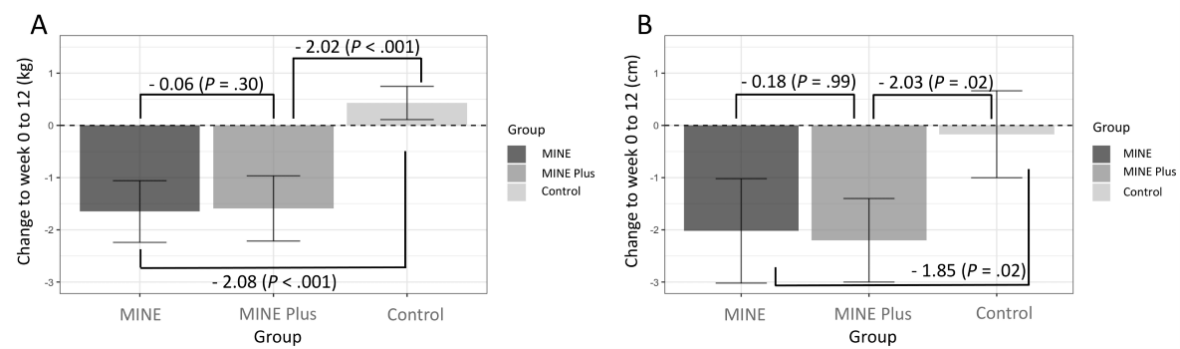
Comparison of (A) body weight and (B) waist circumference interaction effects between time and groups, with the *P* values for the differences between groups from baseline to 12 weeks. MINE: only online education; MINE Plus: online education + tailored feedback.

The interaction between time and group for weight change was not significant from baseline to week 12 after the intervention in the MINE and MINE Plus groups (*P*=.30; Figure 5). The MINE and control groups did not show any interaction effect until week 6 of the intervention (*P*=.11), although there was a significant effect (*P*<.001) compared with week 12 (see Multimedia Appendix 2). The MINE Plus and control groups showed a significant interaction effect when comparing baseline with week 6 and baseline with week 12 (*P*<.001), but this was absent from week 6 to week 12 (*P*=.98).

### Physical Activity Changes

Physical activity was calculated as the percentage of participants meeting the minimum amount of physical activity recommended by the World Health Organization (600 METs/week) [39]. No significant changes in physical activity levels were observed in any group during the postmeasurement and follow-up periods (Table 3).

**Table 3:** Differences in weight, waist circumference (WC), physical activity, and psychological factors between groups (N=60).

Characteristic			MINE^a^ (n=20)		*P* value		MINE Plus^b^ (n=20)		*P* value		Control (n=20)		*P* value
Weight at baseline (kg), mean (SD)			65.82 (7.23)		—^c^		70.64 (13.07)		—		66.2 (7.08)		—
**Weight changes (kg), mean (SD)**													
	Baseline to postintervention	–0.74 (1.96)		.03		–1.87 (1.8)		<.001		0.03 (1.68)		.91	
	Postintervention to follow-up	–0.91 (2.2)		<.001		0.28 (1.72)		.24		0.41 (1.16)		.23	
	Baseline to follow-up	–1.65 (2.64)		<.001		–1.59 (2.79)		<.001		0.43 (1.42)		.27	
WC at baseline (cm), mean (SD)			84.0 (6.21)		—		86.9 (9.81)		—		84.6 (6.54)		—
**WC changes (cm), mean (SD)**													
	Baseline to postintervention	–1.39 (3.57)		.02		–1.95 (2.83)		<.001		–0.09 (2.52)		.93	
	Postintervention to follow-up	–0.63 (3.31)		.29		–0.25 (2.2)		.71		–0.08 (3.13)		.89	
	Baseline to follow-up	–2.02 (4.47)		<.001		–2.2 (3.58)		<.001		–0.17 (3.72)		.82	
Physical activity at baseline (≥600 METs^d^), n (%)			16 (80)		—		13 (65)		—		14 (70)		—
**Physical activity changes (%)^e^, mean (SD)**													
	Baseline to postintervention	–15 (1.83)		.13		0 (2.29)		.60		–5 (1.97)		.60	
	Postintervention to follow-up	10 (2.24)		.31		0 (2.29)		.77		0 (2.29)		.97	
	Baseline to follow-up	5 (1.97)		.81		0 (1.62)		.62		–5 (1.97)		.58	
EAT-26^f,g^ at baseline			6.79 (16.84)		—		10.53 (12.64)		—		6.84 (17.89)		—
**EAT-26 changes, mean (SD)**													
	Baseline to postintervention	4.95 (10.44)		.07		3.3 (10.95)		.22		–0.1 (9.64)		.97	
	Postintervention to follow-up	–0.3 (15.3)		.91		1.25 (8.88)		.64		0.5 (8.26)		.85	
	Baseline to follow-up	4.65 (18.73)		.09		4.55 (12.65)		.09		0.4 (9.68)		.88	
BSQ^h,i^ at baseline, mean (SD)			110.55 (32.96)		—		116.63 (26.48)		—		118.79 (27.56)		—
**BSQ changes, mean (SD)**													
	Baseline to postintervention	–10.45 (29.03)		.02		–10.35 (20.07)		.03		0.7 (18.43)		.88	
	Postintervention to follow-up	–2.25 (13.64)		.62		0.3 (13.29)		.62		2.25 (13.88)		.62	
	Baseline to follow-up	–12.7 (33.67)		.006		–10.05 (16.99)		.03		2.95 (13.9)		.52	
SRAHP^j,k^ at baseline, mean (SD)			70.88 (12.53)		—		74.02 (11.74)		—		72.88 (10.66)		—
**SRAHP changes, mean (SD)**													
	Baseline to postintervention	5.65 (9.83)		.003		7.2 (8.68)		<.001		1.8 (6.83)		.34	
	Postintervention to follow-up	1.1 (9.9)		.56		0.25 (6.54)		.89		2.05 (5.95)		.28	
	Baseline to follow-up	6.75 (12.34)		<.001		7.45 (7.49)		<.001		3.85 (5.63)		.04	

^a^MINE: only online education.

^b^MINE Plus: online education + feedback.

^c^Not applicable.

^d^MET: metabolic equivalent.

^e^The minimum World Health Organization recommended amount of 600 METs minutes per week.

^f^EAT: Eating Attitudes Test.

^g^Higher scores indicate more negative eating attitudes.

^h^BSQ: Body Shape Questionnaire.

^i^Higher scores indicate lower satisfaction.

^j^SRAHP: Self-Rated Abilities of Health Practices.

^k^Higher scores indicate better self-efficacy.

### Psychological Factor Changes

After 12 weeks, the EAT-26 scores increased by 4.65 (SD 18.73; *P*=.09) for the MINE group, 4.55 (SD 12.65; *P*=.09) for the MINE Plus group, and 0.4 (SD 9.68; *P*=.88) for the control group. After 12 weeks, the BSQ scores decreased by –12.7 (SD 33.67; *P*=.006) for the MINE group and –10.05 (SD 16.99; *P*=.03) for the MINE Plus group but increased by 3.85 (SD 5.63; *P*=.04) for the control group, which means the MINE and MINE Plus groups experienced greater satisfaction with their body shape. By 6 weeks, the BSQ scores for the MINE and MINE Plus groups had significantly decreased by –10.45 (SD 29.03; *P*=.02) and –10.35 (SD 20.07; *P*=.03), respectively. However, there was no significant decrease from 6 weeks to 12 weeks, the period during which there was no intervention. The SRAHP scores increased by 6.75 (SD 12.34; *P*<.001) for the MINE Plus group and 7.45 (SD 7.49; *P*<.001) for the control group after 12 weeks. After the intervention, the SRAHP scores for both the MINE and MINE Plus groups increased significantly, by 5.65 (SD 9.83; *P*=.003) and 7.2 (SD 8.68; *P*<.001), respectively, but did not increase significantly until 12 weeks after the intervention.

## Discussion

### Principal Findings

This study confirmed changes in weight loss and psychological factors for women with overweight and obesity aged in their 20s and 30s through a web-based, weight-related, behavioral change program. Both the MINE group, who received only web-based education, and the MINE Plus group, who received web-based education and feedback, showed significant weight loss. Regarding psychological factors, significant positive changes were observed only in body shape satisfaction and health management self-efficacy. 

Of the 60 participants in this study, 54 completed the postintervention and follow-up measurements, resulting in a dropout rate of ~10%. Mobile- or web-based, health-related interventions are usually reported to have high dropout rates [25]. According to a systematic literature review by Kelders et al [40], the median duration of web-based health interventions was 10 weeks, and the mean adherence rate for web-based trial participants was 55%. However, the proportions of participants who received the education and submitted quizzes were 68% in the MINE group and 84% in the MINE Plus group (Multimedia Appendix 3). This showed higher compliance than that observed in previous studies, due to continuous motivation and the accessibility of educational content. Therefore, this study shows that even web-based experimental studies can have low dropout rates.

### Comparison With Previous Work

Compared with previous studies, a study by Baer et al [23] showed an average weight loss of –3.1 (95% CI –3.7 to –2.5) kg over 12 months in an integrated intervention group with online programs and health managers. In addition, studies based on other online platforms and coaching programs showed an average weight loss of –1.57 (95% CI –1.92 to –1.22) kg over 24 weeks [15]. In this study, the average 12-week loss in the intervention groups was –1.65 (SD 2.64) kg in the MINE group and –1.59 (SD 2.79) kg in the MINE Plus group, which was a significant loss compared with the weight difference observed in previous studies.

In our study, weight loss and lifestyle changes were documented through app use records, feedback, and satisfaction surveys; however, objective indicators could not be confirmed. A lack of power, insufficient time to produce results, and insufficient sensitivity of measurement tools to detect minor differences could be reasons for no differences in physical activity or cardiovascular outcomes [21]. To increase physical activity, it is considered essential to identify individual vulnerabilities through “just-in-time” feedback and to set goals suitable for participants, taking into consideration the available time and circumstances [41]. It also requires specific guidance and clarification on when, where, and how to act [42]. Considering this situation, interventions and elements that can increase physical activity and make it habitual should be further developed.

There was no significant difference in weight reduction in the MINE Plus group in the absence of the intervention. The group who received feedback showed a higher rate of weight loss until the intervention period, but it is likely that independence decreased after the intervention ended due to the absence of coaching. Participants may experience various problems such as anxiety and decreased self-confidence if they receive feedback for a certain period and then abruptly discontinue it for a short period [43,44]. In contrast, it seemed that the MINE group showed a continuous form of weight loss by managing goal setting based on the educational content. Therefore, it is necessary to establish a more effective feedback methodology when mediating feedback and proceed to provide feedback until the participants are independent and weight management is achieved through long-term feedback. However, considering that the waist circumference of the MINE Plus group decreased more than that of the MINE group over the entire period, it is inferred that more effective health management was conducted.

A previous study confirmed the positive effect of body shape satisfaction using Internet-based mindfulness interventions [45]. In this study also, there was a significant improvement in body shape satisfaction as the education program provided strategies to the participants on how to cope with social stigma or stress, similar to mindfulness strategies. Finally, it seems that continuous motivation and encouragement were helpful through tailored feedback.

In a study confirming the relationship between weight loss and health self-efficacy in young adults, self-efficacy increased through weight management education and showed effective results in promoting weight loss [46]. In this study, participants managed their weight with methods that could set and achieve goals through education. It showed that providing goals through feedback, complimenting them, and encouraging them were helpful. However, no other positive results could be confirmed after week 6. Previous research has indicated that the effects of tailored feedback are controversial, with effectiveness diminishing over extended periods of observation, showing no significant difference from groups who did not receive intervention [47,48]. The provision of goals is significantly correlated with self-efficacy, which leads to the creation of sustainable self-set goals [49]. However, it appears that, after the intervention and education period, the participants' self-efficacy and self-set goal development did not expand without being given goals. Therefore, there is a need for interventions in which participants can continue to be motivated and achieve goals.

Psychological factors for weight loss identified for young women in this study were to treat obesity when young and relieve psychological pressure through healthy weight loss. Obesity treatment should proceed in a healthy way that prevents access to compulsive and distorted knowledge and can be self-applied [50]. Therefore, the program was organized in a way to empower the participants to improve their habits and perceptions through education rather than by forced intervention. This study progressed with education and feedback based on various theories, including the social cognitive theory. Although it is difficult to determine which theories and methods worked best in the program, the approach of integrated theory will be more critical in health education [21]. Furthermore, future research should investigate and compare the effectiveness of various delivery methods in online programs to identify which theories and strategies produce the most substantial outcomes for participants or patients [51]. Furthermore, collective and institutional efforts should be accompanied at the national level to improve long-term weight management and dietary and activity environments [52].

### Strengths and Limitations

This study has several strengths. First, the program was constructed by approaching the treatment of obesity in a multifactorial manner rather than by considering just one factor. Tools such as nutrition, physical activity, psychological and medical knowledge education, and tailored feedback were used to improve lifestyle, and the program was designed so that participants could improve themselves. Second, this approach can reduce time, expenses, and human resources. Since this program is only conducted online, participants can proceed with web-based education and feedback according to their preferred place and time. Furthermore, educational videos can be reused later. Feedback also allows participants and moderators to participate at any time and place of their choice. As they turned on the camera and communicated in real time, the participants could see each other’s facial expressions, understand emotions, and build bonds. Third, existing behavioral change programs were used to supplement and construct obesity treatment strategy elements in the program. Various behavioral change factors, such as goal setting, self-monitoring, and self-control, which are essential and proven effective, were continuously added to the educational content and feedback to maximize effectiveness. However, there are several limitations to this study. First, there was no long-term follow-up of the participants. Obesity is an area that requires continuous management, and it is necessary to set a period for long-term follow-up. According to the meta-analysis by Beleigoli et al [17], web-based digital interventions have shown effectiveness in the short term but not in the long term. Therefore, for long-term management, it seems necessary to combine offline management with digital interventions. Second, the use of various objective indicators was insufficient. The indicators for physical activity and psychological factors in this study were in the form of questionnaires. Therefore, more objective data should be collected through the observation of physical activity using accelerometers or various blood and biomarker data. Third, the nutritional information collected through self-monitoring was not evaluated due to measurement bias. It appears that future research will require accurate collection and analysis of nutritional information. Fourth, only university undergraduates, graduate students, and faculty members participated in this study. From a demographic perspective, most of them were highly educated participants. Therefore, the data from this program cannot be generalized to all other young adult women in the real world. Last, there was no significant change in physical activity because of the spread of COVID-19 in South Korea during the experimental period.

### Conclusions

This study demonstrated the efficacy of a web-based education program, with and without tailored feedback, in promoting weight loss and enhancing psychological well-being through self-managed diet and exercise modifications. The 2 groups who received the intervention experienced significant weight loss over time, yet the magnitude of weight reduction varied across periods. Although improvements were observed in various psychological factors, these psychological improvements did not persist in the absence of the intervention. This indicates a need to integrate social support, incentives, or motivation in future digital health interventions and underscores the importance of interdisciplinary research in this field.

## Data Availability

The data set generated and analyzed in this study will be available from the corresponding author after the study at reasonable request.

## Appendix

Multimedia Appendix 1 Web-based education program and tailored feedback satisfaction.

Multimedia Appendix 2 Supplementary table (Effects of interaction between time and groups).

Multimedia Appendix 3 Quiz submission rate.

Multimedia Appendix 4 CONSORT-EHEALTH checklist.

## Abbreviations

BP: blood pressure
BSQ: Body Shape Questionnaire
EAT: Eating Attitudes Test
GPAQ: Global Physical Activity Questionnaire
MET: metabolic equivalent
SRAHP: Self-rated Abilities for Health Practices scale
