# Supplementary material for: Effects of a Web-based Weight Management Education Program on Various Factors for Overweight and Obese Women: Randomized Controlled Trial
Source: JMIR Cardio. 2024 Apr 18;8:e42402. doi: 10.2196/42402 (PMC11066746; doi:10.2196/42402)
Supplement: Multimedia Appendix 1 [file cardio_v8i1e42402_app1.docx]

Multimedia Appendix 1. Web-based education program and tailored feedback satisfaction.

'MINE' refers to the group that only received online education as an intervention. 'MINE +' refers to the group that received both online education and tailored feedback as interventions. The questionnaire is on a 5-point Likert scale. The higher the score, the higher the satisfaction.
