# Supplementary material for: Effects of a Web-based Weight Management Education Program on Various Factors for Overweight and Obese Women: Randomized Controlled Trial
Source: JMIR Cardio. 2024 Apr 18;8:e42402. doi: 10.2196/42402 (PMC11066746; doi:10.2196/42402)
Supplement: Multimedia Appendix 2 [file cardio_v8i1e42402_app2.docx]

Appendix 2. Supplementary table (Effects of interaction between time and groups)

| **Body Weight** |  | **MINE^a^ × MINE** |  |  | **MINE+ × MINE+^b^** |  |  | **Control × Control** |  |  |
| --- | --- | --- | --- | --- | --- | --- | --- | --- | --- | --- |
| **Time** | pre-post | pre-follow up | post-follow up | pre-post | pre-follow up | post-follow up | pre-post | pre-follow up | post-follow up |  |
| ***P* -value** | .03 | <.001 | .007 | <.001 | <.001 | .24 | .91 | .23 | .27 |  |
|  |  | **MINE × MINE+** |  |  | **MINE+ × Control** |  |  | **MINE+ × Control** |  |  |
| **Group:Time** | pre-post | pre-follow up | post-follow up | pre-post | pre-follow up | post-follow up | pre-post | pre-follow up | post-follow up |  |
| ***P* -value** | .08 | .30 | .006 | .11 | <.001 | .007 | <.001 | <.001 | .98 |  |
|  |  |  |  |  |  |  |  |  |  |  |
| **BMI** |  | **MINE × MINE** |  |  | **MINE+ × MINE+** |  |  | **Control × Control** |  |  |
| **Time** | pre-post | pre-follow up | post-follow up | pre-post | pre-follow up | post-follow up | pre-post | pre-follow up | post-follow up |  |
| ***P* -value** | .06 | <.001 | .03 | <.001 | <.001 | .27 | .79 | .22 | .34 |  |
|  |  | **MINE × MINE+** |  |  | **MINE+ × Control** |  |  | **MINE+ × Control** |  |  |
| **Group:Time** | pre-post | pre-follow up | post-follow up | pre-post | pre-follow up | post-follow up | pre-post | pre-follow up | post-follow up |  |
| ***P* -value** | .04 | .79 | .02 | .12 | <.001 | .03 | <.001 | <.001 | .94 |  |
|  |  |  |  |  |  |  |  |  |  |  |
| **VFA** |  | **MINE × MINE** |  |  | **MINE+ × MINE+** |  |  | **Control × Control** |  |  |
| **Time** | pre-post | pre-follow up | post-follow up | pre-post | pre-follow up | post-follow up | pre-post | pre-follow up | post-follow up |  |
| ***P* -value** | .048 | <.001 | <.001 | .001 | <.001 | .45 | .72 | .40 | .23 |  |
|  |  | **MINE × MINE+** |  |  | **MINE+ × Control** |  |  | **MINE+ × Control** |  |  |
| **Group:Time** | pre-post | pre-follow up | post-follow up | pre-post | pre-follow up | post-follow up | pre-post | pre-follow up | post-follow up |  |
| ***P* -value** | .48 | .07 | .01 | .10 | <.001 | .04 | .01 | .04 | .70 |  |
|  |  |  |  |  |  |  |  |  |  |  |
| **Pfat** |  | **MINE × MINE** |  |  | **MINE+ × MINE+** |  |  | **Control × Control** |  |  |
| **Time** | pre-post | pre-follow up | post-follow up | pre-post | pre-follow up | post-follow up | pre-post | pre-follow up | post-follow up |  |
| ***P* -value** | .06 | <.001 | .03 | .005 | <.001 | .23 | .60 | .049 | .15 |  |
|  |  | **MINE × MINE+** |  |  | **MINE+ × Control** |  |  | **MINE+ × Control** |  |  |
| **Group:Time** | pre-post | pre-follow up | post-follow up | pre-post | pre-follow up | post-follow up | pre-post | pre-follow up | post-follow up |  |
| ***P* -value** | .51 | .95 | .47 | .34 | .14 | .59 | 0.11 | 0.15 | 0.86 |  |
|  |  |  |  |  |  |  |  |  |  |  |
| **WC** |  | **MINE × MINE** |  |  | **MINE+ × MINE+** |  |  | **Control × Control** |  |  |
| **Time** | pre-post | pre-follow up | post-follow up | pre-post | pre-follow up | post-follow up | pre-post | pre-follow up | post-follow up |  |
| ***P* -value** | .02 | <.001 | .29 | .001 | <.001 | .71 | .93 | .82 | .89 |  |
|  |  | **MINE × MINE+** |  |  | **MINE+ × Control** |  |  | **MINE+ × Control** |  |  |
| **Group:Time** | pre-post | pre-follow up | post-follow up | pre-post | pre-follow up | post-follow up | pre-post | pre-follow up | post-follow up |  |
| ***P* -value** | .61 | .99 | .62 | .10 | .02 | .51 | .03 | .02 | .87 |  |
|  |  |  |  |  |  |  |  |  |  |  |
| **SBP** |  | **MINE × MINE** |  |  | **MINE+ × MINE+** |  |  | **Control × Control** |  |  |
| **Time** | pre-post | pre-follow up | post-follow up | pre-post | pre-follow up | post-follow up | pre-post | pre-follow up | post-follow up |  |
| ***P* -value** | .41 | .87 | .51 | .51 | .57 | .92 | .03 | .60 | .10 |  |
|  |  | **MINE × MINE+** |  |  | **MINE+ × Control** |  |  | **MINE+ × Control** |  |  |
| **Group:Time** | pre-post | pre-follow up | post-follow up | pre-post | pre-follow up | post-follow up | pre-post | pre-follow up | post-follow up |  |
| ***P* -value** | .91 | .78 | .69 | .34 | .80 | .48 | .41 | .28 | .27 |  |
|  |  |  |  |  |  |  |  |  |  |  |
| **DBP** |  | **MINE × MINE** |  |  | **MINE+ × MINE+** |  |  | **Control × Control** |  |  |
| **Time** | pre-post | pre-follow up | post-follow up | pre-post | pre-follow up | post-follow up | pre-post | pre-follow up | post-follow up |  |
| ***P* -value** | .86 | .72 | .59 | .64 | .40 | .72 | <.001 | .03 | .18 |  |
|  |  | **MINE × MINE+** |  |  | **MINE+ × Control** |  |  | **MINE+ × Control** |  |  |
| **Group:Time** | pre-post | pre-follow up | post-follow up | pre-post | pre-follow up | post-follow up | pre-post | pre-follow up | post-follow up |  |
| ***P* -value** | .64 | .74 | .90 | .02 | .07 | .57 | .005 | .03 | .49 |  |
|  |  |  |  |  |  |  |  |  |  |  |
| **HR** |  | **MINE × MINE** |  |  | **MINE+ × MINE+** |  |  | **Control × Control** |  |  |
| **Time** | pre-post | pre-follow up | post-follow up | pre-post | pre-follow up | post-follow up | pre-post | pre-follow up | post-follow up |  |
| ***P* -value** | .92 | .96 | .89 | .77 | .70 | .50 | .11 | .87 | .15 |  |
|  |  | **MINE × MINE+** |  |  | **MINE+ × Control** |  |  | **MINE+ × Control** |  |  |
| **Group:Time** | pre-post | pre-follow up | post-follow up | pre-post | pre-follow up | post-follow up | pre-post | pre-follow up | post-follow up |  |
| ***P* -value** | .89 | .81 | .71 | .28 | .88 | .35 | .34 | .70 | .58 |  |
|  |  |  |  |  |  |  |  |  |  |  |
| **Physical activity**  **changes** |  | **MINE × MINE** |  |  | **MINE+ × MINE+** |  |  | **Control × Control** |  |  |
| **Time** | pre-post | pre-follow up | post-follow up | pre-post | pre-follow up | post-follow up | pre-post | pre-follow up | post-follow up |  |
| ***P* -value** | .13 | .56 | .31 | .61 | .62 | .77 | .60 | .58 | .96 |  |
|  |  | **MINE × MINE+** |  |  | **MINE+ × Control** |  |  | **MINE+ × Control** |  |  |
| **Group:Time** | pre-post | pre-follow up | post-follow up | pre-post | pre-follow up | post-follow up | pre-post | pre-follow up | post-follow up |  |
| ***P* -value** | .14 | .44 | .46 | .14 | .43 | .48 | .98 | .98 | .76 |  |
|  |  |  |  |  |  |  |  |  |  |  |
| **EAT-26^c,d^** |  | **MINE × MINE** |  |  | **MINE+ × MINE+** |  |  | **Control × Control** |  |  |
| **Time** | pre-post | pre-follow up | post-follow up | pre-post | pre-follow up | post-follow up | pre-post | pre-follow up | post-follow up |  |
| ***P* -value** | .07 | .09 | .91 | .22 | .09 | .64 | .97 | .88 | .85 |  |
|  |  | **MINE × MINE+** |  |  | **MINE+ × Control** |  |  | **MINE+ × Control** |  |  |
| **Group:Time** | pre-post | pre-follow up | post-follow up | pre-post | pre-follow up | post-follow up | pre-post | pre-follow up | post-follow up |  |
| ***P* -value** | .67 | .98 | .69 | .19 | .27 | .83 | .37 | .28 | .84 |  |
|  |  |  |  |  |  |  |  |  |  |  |
| **BSQ^e,f^** |  | **MINE × MINE** |  |  | **MINE+ × MINE+** |  |  | **Control × Control** |  |  |
| **Time** | pre-post | pre-follow up | post-follow up | pre-post | pre-follow up | post-follow up | pre-post | pre-follow up | post-follow up |  |
| ***P* -value** | .02 | .006 | .62 | .03 | .03 | .95 | .88 | .52 | .62 |  |
|  |  | **MINE × MINE+** |  |  | **MINE+ × Control** |  |  | **MINE+ × Control** |  |  |
| **Group:Time** | pre-post | pre-follow up | post-follow up | pre-post | pre-follow up | post-follow up | pre-post | pre-follow up | post-follow up |  |
| ***P* -value** | .99 | .68 | .69 | .09 | .02 | .49 | .09 | .047 | .76 |  |
|  |  |  |  |  |  |  |  |  |  |  |
| **SRAHP^g,h^** |  | **MINE × MINE** |  |  | **MINE+ × MINE+** |  |  | **Control × Control** |  |  |
| **Time** | pre-post | pre-follow up | post-follow up | pre-post | pre-follow up | post-follow up | pre-post | pre-follow up | post-follow up |  |
| ***P* -value** | .003 | <.001 | .56 | <.001 | <.001 | .89 | .34 | .04 | .28 |  |
|  |  | **MINE × MINE+** |  |  | **MINE+ × Control** |  |  | **MINE+ × Control** |  |  |
| **Group:Time** | pre-post | pre-follow up | post-follow up | pre-post | pre-follow up | post-follow up | pre-post | pre-follow up | post-follow up |  |
| ***P* -value** | .56 | .79 | .75 | .15 | .28 | .72 | .04 | .18 | .89 |  |
|  |  |  |  |  |  |  |  |  |  |  |
| Abbreviations: BMI, body mass index; VFA, visceral fat area; Pfat, percentage body fat; WC, waist circumference;  SBP, systolic blood pressure; DBP, diastolic blood pressure; HR, heart rate  ^a^MINE: only online education.  ^b^MINE Plus: online education + tailored feedback.  ^c^EAT-26: Eating Attitudes Test-26 (Korean version).  ^d^Higher scores indicate more negative eating attitudes.  ^e^BSQ: Body Shape Questionnaire (Korean version).  ^f^Higher scores indicate lower satisfaction.  ^g^SRAHP: Self-Rated Abilities of Health Practices (Korean version).  ^h^Higher scores indicate better self-efficacy. | | | | | | | | | |  |
|  |  |  |  |  |  |  |  |  |  |  |
