# Supplementary material for: Effects of a Web-based Weight Management Education Program on Various Factors for Overweight and Obese Women: Randomized Controlled Trial
Source: JMIR Cardio. 2024 Apr 18;8:e42402. doi: 10.2196/42402 (PMC11066746; doi:10.2196/42402)
Supplement: Multimedia Appendix 3 [file cardio_v8i1e42402_app3.doc]

Multimedia Appendix 3. Quiz submission rate

| No. | 1 | 2 | 3 | 4 | 5 | 6 | 7 | 8 |  |
| --- | --- | --- | --- | --- | --- | --- | --- | --- | --- |
| **MINE^a^** | 100 | 100 | 100 | 94.1 | 94.1 | 94.1 | 88.2 | 64.7 |  |
| **MINE+^b^** | 94.1 | 88.2 | 94.1 | 88.2 | 94.1 | 94.1 | 82.4 | 88.2 |  |
| No. | 9 | 10 | 11 | 12 | 13 | 14 | 15 | 16 | Average |
| **MINE** | 58.8 | 58.8 | 58.8 | 41.2 | 41.2 | 35.3 | 29.4 | 35.3 | **68.4** |
| **MINE+** | 70.6 | 82.4 | 88.2 | 88.2 | 88.2 | 70.6 | 70.6 | 70.6 | **84.6** |

Data were indicated as the percentage of all participants who watched the education video and submitted the quiz.

^a^MINE: only online education.

^b^MINE Plus: online education + tailored feedback.
